# A Case of Pelvic Hæmatocele

**Published:** 1888-03

**Authors:** Virgil O. Hardon

**Affiliations:** Professor of Obstetrics and Diseases of Women and Children, Atlanta Medical College, Atlanta, Ga.


### A CASE OF PELVIC HÆMATOCELE.

BY VIRGIL O. HARDON, M. D., PROFESSOR OF OBSTETRICS AND DISEASES OF WOMEN AND CHILDREN, ATLANTA MEDICAL COLLEGE, ATLANTA, GA.

Mrs. F. N. M., white, married, aged 36, the mother of three children, the youngest six years of age, was suddenly seized with cramps about noon, on the 31st of March, 1887, while engaged in her usual household avocations. Her previous health had been good, but her menses had not appeared for six weeks. She had not suspected pregnancy. As her pains did not yield to domestic remedies, I was summoned to her relief. On my arrival I found her lying on the bed, suffering intensely from pains in the lower portion of the abdomen and in a state of complete collapse. The skin was pallid and cold, the lips bloodless, the radial pulse feeble but rapid, temperature in the axilla 97.6. The pain was located in the hypogastric and iliac regions, and was so severe as to elicit continuous cries, although the patient was a woman of a considerable degree of fortitude. The bowels had acted in the morning and the bladder had been emptied about a half hour before the commencement of the attack. Palpation over the seat of pain showed slight tenderness on pressure, while percussion gave an area of dullness extending across the abdomen and upward to a line midway between the pubes and the umbilicus. Above this line there was some tympanites. There was slight nausea. Vaginal examination showed the uterus to be low in the pelvis, the cervix soft, the os uteri patulous. Behind the cervix could be felt a rounded, bulging, fluctuating tumor, which was forced further downward into the vagina by pressure above the pubes. By the rectum this fluctuating mass could be more distinctly felt, extending upward as far as the finger could reach.

A hypodermic injection of one-half a grain of morphine was given, which only partially relieved the pain. Whisky was administered by the mouth freely. Hot bricks were placed to the extremities and to the sides of the body and a mustard-plaster over the heart. In about six hours reaction was established; the pulse became stronger, the surface of the body warmer and the axillary temperature normal. The pain in the abdomen continued, but was controlled by morphine administered hypodermically. An exclusive milk diet was ordered and rigid quietude enjoined.

*April 1.*—Patient was unable to sleep on account of pain. The abdomen was swollen, tympanitic and excessively painful and tender to the level of the diaphragm. Respiration painful, rapid and superficial. Pulse 110, temp. 101.2. Skin hot and dry, tongue coated, patient complains of thirst. Micturition frequent and accompanied by lancinating pains.

From this time the pulse and temperature steadily rose, the highest point being reached on the third day, when they were respectively 144 and 104.6. The tenderness and distention of the abdomen were extreme. Pain was constant, and on the third day there was mild delirium. There was no vomiting at any time and no nausea after the first day. On the fourth day the symptoms began to abate, and the bowels were freely moved by a dose of sulphate of magnesia. The treatment consisted of morphine in sufficient quantities to relieve pain, and stimulants and milk pushed to the utmost limit of the patient's ability to retain them. By the tenth day the pulse and temperature were normal. During all this time the patient had been kept strictly confined to the recumbent position.

The abatement of the acute inflammatory symptoms left the patient much exhausted. A tonic of citrate of iron and quinine in sherry wine was therefore prescribed with nourishing diet. Vaginal examination showed the pelvis to be completely filled with a hard, resisting mass which pressed upon the rectum, projected in a rounded tumor into the posterior fornix of the vagina, and surrounded and immovably fixed the uterus. The patient complained of a sense of weight and pressure in the pelvis. There was only slight tenderness upon pressure in the roof of the vagina.

On the 13th of April there was another attack of sudden collapse, less severe, however, than the previous one. This was followed by a renewal of the peritonitis, during which the pulse rose to 126 and the temperature to 102. The inflammatory symptoms lasted for four days and were treated in the same manner as before.

After the subsidence of this attack the patient gradually regained her strength, and on the 2nd of May was allowed to sit up. The abdomen remained tender to pressure for about three weeks after this date. The sense of fullness and pressure in the pelvis gradually diminished. The hard mass in the pelvis softened down and diminished in size until only a rounded tumor as large as a small orange could be felt behind the uterus and pressing on the rectum. The menses appeared on the 4th of May and were accompanied by an unusual amount of pain. In quantity, color and duration the flow was normal.

By the middle of May the patient had regained her usual health and was able to resume her customary household duties. Some tenderness of the hypogastric region still remained and a hard mass could be felt in Douglas' pouch. On the 29th of May there was passed *per rectum* about a teacupful of black, offensive and decomposed blood mixed with pus. Vaginal examination on the next day showed that the mass behind the uterus had entirely disappeared.

From that time the patient enjoyed uninterrupted good health until her departure from the city on the 28th of June. The pelvic symptoms entirely disappeared and she gained rapidly in weight.

The symptoms upon which was based the diagnosis of pelvic hæmatocele in this case were:

1st. The sudden and profound collapse.

2nd. The subsequent acute peritonitis.

3d. The existence of an effusion into the pelvic cavity which soon solidified into a hard, resisting mass.

4th. The passage from the bowel of a quantity of decomposed blood, mixed with pus, coincident with the disappearance of the remains of the pelvic effusion.

